# Causal factors and configuration paths for chemical occupational accidents: grounded theory and fsQCA analysis of 154 cases in China

**DOI:** 10.3389/fpubh.2025.1713532

**Published:** 2026-01-12

**Authors:** Yuemeng Wang, Congju Zheng, Li Wang

**Affiliations:** 1 Business School, Yangzhou University, Yangzhou, China; 2 School of Management, Minzu University of China, Beijing, China; 3 School of Public Policy and Management, China University of Mining and Technology, Xuzhou, China

**Keywords:** causal factors, chemical occupational accidents, configuration paths, FsQCA, latent-active accident causation model (LA-ACM)

## Abstract

**Background:**

Chemical occupational accidents remain a critical threat to worker safety in China, but existing research has often failed to capture the complex, nonlinear, multi-factor coupling mechanisms underlying accident causation.

**Methods:**

This mixed-methods study analyzed 154 Chinese chemical occupational accident cases by integrating grounded theory and fuzzy-set qualitative comparative analysis (fsQCA). Employing the three-stage coding process of grounded theory (open, axial, and selective) to identify key causal factors, the study then used fsQCA to explore the configuration paths leading to both high- and low-severity accidents.

**Results and discussion:**

Grounded theory analysis identified 55 preliminary categories, 16 main categories, and 5 overarching core categories: safety culture, safety management system, safety capability, safety behavior, and worksite condition. These categories form the basis of the proposed Latent-Active Accident Causation Model (LA-ACM), which classifies them into latent conditions and active failures. FsQCA revealed four high-severity paths, namely safety culture-driven, safety management-dominated, safety capability-deficient, and multi-triggered, and two low-severity paths, including safety behavior-induced and worksite condition-triggered. Accordingly, a two-tier risk prevention framework was proposed: the “latent condition rectification layer” targets systemic gaps, and the “active failure interception layer” addresses on-site risks. This study underscores the theoretical implications for upstream safety governance and offers practical strategies to reduce the frequency and severity of chemical occupational accidents.

## 1 Introduction

According to the report *The Global Chemical Industry: Catalyzing Growth and Addressing Our World′s Sustainability Challenges* released by the International Council of Chemical Associations (ICCA), the chemical industry is estimated to have contributed $5.7 trillion to the global Gross Domestic Product (GDP), accounting for 7% of the global GDP, and has provided 120 million jobs worldwide (1). However, its inherent characteristics, including complex production processes, extensive use of hazardous chemicals, and high operational risks, pose persistent threats to occupational safety (2, 3). Previous estimates published by the International Labour Organization (ILO) have found that over 2.78 million workers die globally each year due to their working conditions (4) and that exposure to hazardous substances claims the lives of almost 1 million workers (5). This translates to at least one worker dying every 30 s due to occupational chemical exposure (6). The gravity of occupational safety risks extends from macro-level statistics to micro-level implementation. Malta et al. (2024) have identified specific failures—the widespread neglect of recording and verifying Personal Protective Equipment (PPE) use and the dependence on formalistic safety documentation—as prevalent yet underestimated vulnerabilities in the accident prevention framework (7). Beyond casualties and immediate economic losses, chemical occupational accidents may lead to long-term environmental contamination and social disruption from hazardous substance leaks (8, 9). The tension between the chemical industry’s socioeconomic value and its safety risks underscores the urgent need for stronger safety prevention strategies worldwide (10, 11).

The evolution of the chemical occupational safety management system has witnessed a paradigm shift from passive post-accident response to proactive systematic prevention. Early research focused primarily on technical standards and equipment-based safeguards to mitigate the consequences of an accident (12, 13), with limited attention to non-technical factors. A pivotal turning point came in 1992, when the U.S. Occupational Safety and Health Administration (OSHA) introduced the Process Safety Management (PSM) standard (29 CFR 1910.119), establishing a framework centered on process hazard analysis (PHA) and mechanical integrity (MI) (14). While PSM reduced U.S. chemical process accidents by 40% between 1998 and 2018, its overemphasis on technical equipment overlooked the systemic impacts of human behavior and organizational deficiencies (15, 16). This “hard over soft” bias has limited the framework’s effectiveness in analyzing complex, multifactorial accidents—a weakness increasingly evident in real-world chemical occupational accident cases. In contrast to this technically oriented framework, the EU’s Seveso III Directive (2012) exemplified a pivotal shift by formally integrating organizational and human factors into a comprehensive framework, thereby championing an integrated approach to risk prevention and a heightened regulatory focus on systemic safety (17).

Advancements in accident investigation techniques and critical reflections on major disasters have since highlighted the central role of human error and organizational failures in chemical accidents (18). Building on Reason’s (1990) Swiss Cheese Model (19), Shappell and Wiegmann (2004) developed the Human Factors Analysis and Classification System (HFACS), a structured framework for analyzing human factors in accidents (20). Wang et al. (2020) adapted this to HFACS-CSMEs for small and medium-sized chemical enterprises, finding that organizational deficiencies accounted for 76% of unsafe supervision behavior (21). A meta-analysis by Yalcin et al. (2023) further confirmed that 68% of chemical maintenance accidents are due to human error (22). Despite these insights, HFACS suffers from two key limitations: its recognition rate for specific factors lags far behind their actual occurrence (23), and it tends to analyze human factors in isolation, failing to capture dynamic interactions within human-machine-environment systems (24).

In recent years, systems theory models have introduced a new analytical paradigm to safety research. Leveson’s (2004) Systems Theory Accident Model and Process (STAMP) breaks free from traditional linear causal thinking, framing accidents as failures in multi-level control structures including technology, organization, and human factors (25). When STAMP is integrated with methods such as fuzzy DEMATEL, it enables nonlinear, dynamic accident analysis and the identification of hidden risks (26, 27). For instance, Sun et al. (2022) applied STAMP to chemical process safety, developing a dynamic resilience assessment method for diesel hydrogenation systems (28). However, STAMP and similar system models face methodological and practical constraints: First, analyzing complex single cases is time-intensive, making large-sample studies cost-prohibitive (29). Second, ambiguous system boundaries and reliance on subjective judgment risk expanding the analysis scope arbitrarily or omitting critical external factors, thereby undermining the objectivity and comparability of the results (30). These limitations restrict their applicability to industry-level accident causation research.

To address these gaps, this study integrated grounded theory and fuzzy-set qualitative comparative analysis (fsQCA) to analyze 154 cases of chemical occupational accidents in China from 2010 to 2023. The primary goal is to identify multi-dimensional causal factors and complex causal pathways underlying these accidents, providing theoretical support and practical guidance for advancing “upstream” occupational safety governance in the chemical industry. The innovations are threefold: (1) Theoretical Construction: Through three-stage coding, five overarching core categories (safety culture, safety management system, safety capability, safety behavior, worksite condition) were extracted to develop the Latent-Active Accident Causation Model (LA-ACM); (2) Mechanism Analysis: FsQCA was used to explore configuration paths stratified by accident severity, which elucidated the distinct trigger patterns for high- and low-severity incidents, thus contributing to a more nuanced hierarchical accident causation framework; (3) Practical Strategy: A two-tier risk prevention framework, comprising a “latent condition rectification layer” to mitigate systemic gaps and an “active failure interception layer” to intercept on-site risks, was proposed to offer targeted strategies for precise accident prevention, in the chemical industry.

The remainder of this paper is structured as follows: Section 2 outlines the research design, including methods and data sources; Section 3 presents grounded theory-based coding analysis and constructs the LA-ACM; Section 4 uses fsQCA to dissect multi-configuration pathways for accidents of varying severity; Section 5 summarizes key findings, proposes policy recommendations, and outlines future research directions.

## 2 Research design

To systematically unpack the causal mechanisms of chemical occupational accidents, this study adopts a mixed-methods approach integrating grounded theory and fsQCA, applied to 154 cases of chemical occupational accidents in China (2010–2023). The research proceeds in two sequential phases: First, grounded theory-based three-stage coding is used to identify 55 preliminary categories, 16 main categories and 5 overarching core categories, which are utilized for the construction of LA-ACM; second, fsQCA is employed to conduct necessity tests and conditional configuration analysis, with a focus on revealing the synergistic effects of multiple causal factors, causal chain relationships, and differentiated path patterns. This dual-method design addresses the complexity of accident causation in the chemical industry, providing a novel theoretical framework and empirical basis for chemical occupational safety governance.

### 2.1 Research methods

#### 2.1.1 Grounded theory

**Figure 1:**
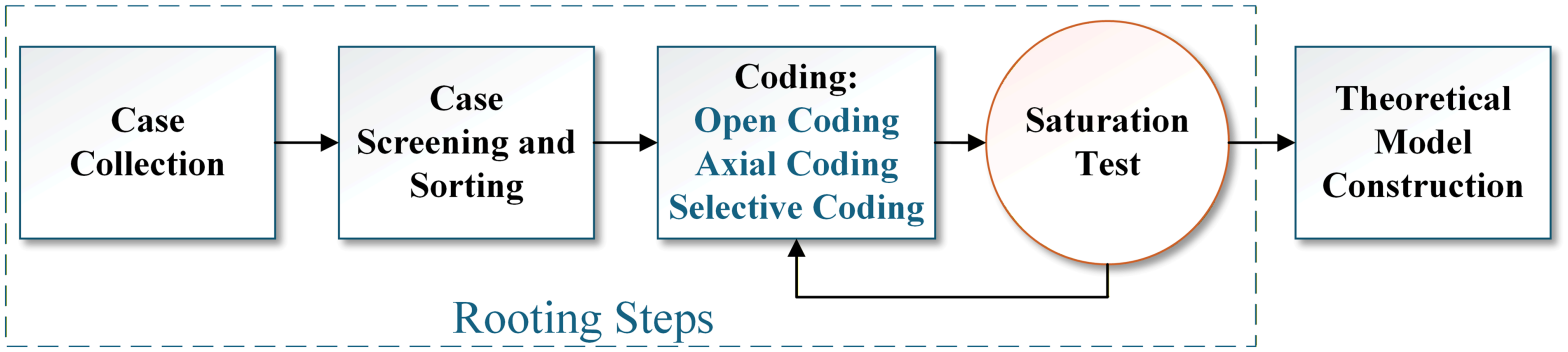
Rooting steps.

Grounded theory is a qualitative research method designed to construct theoretical models through systematic data collection and inductive analysis (31). It emphasizes deriving concepts and categories directly from raw data, rather than testing preconceived hypotheses, allowing researchers to transcend surface-level observations and uncover hidden structural relationships and patterns within complex datasets (32). In this study, grounded theory was strictly applied to code and categorize chemical occupational accident cases following a three-stage procedure: during open coding, initial concepts were extracted from accident investigation reports, resulting in the identification of preliminary categories; axial coding then aggregated these preliminary categories into main categories based on their thematic relevance; and selective coding further integrated the main categories into overarching core categories, which served as the theoretical backbone of the accident causation model. To validate the completeness of the coding process, a saturation test was conducted; no new categories or relationships emerged upon analyzing the independent validation subsample, confirming that the theoretical framework was sufficiently comprehensive to capture the key dimensions of accident causation (see Figure 1).

#### 2.1.2 Fuzzy set qualitative comparative analysis

Fuzzy-set qualitative comparative analysis (fsQCA) addresses limitations of traditional research methods: it overcomes the reliance on large samples in conventional quantitative analysis and the focus on single-case depth in qualitative research (33). By analyzing configurations of variables (rather than isolated factors), fsQCA can explore how combinations of conditions jointly influence an outcome—critical for understanding complex, multi-factor phenomena like accident severity. This method is particularly suited to identifying “causal recipes” that lead to different outcome states (34), enabling the development of tailored risk management strategies.

For this study, fsQCA was employed to analyze the causes among chemical occupational accidents across different severity levels, following three key steps: first, in variable operationalization, condition variables (derived from the five overarching core categories of the LA-ACM) and outcome variables (accident severity, categorized as high or low) were calibrated using fuzzy-set scores based on textual data from the coded accident cases; second, in necessity analysis, univariate tests were conducted to determine whether any single condition was necessary for high-severity or low-severity accidents; third, in configuration analysis, truth tables were constructed to identify all logically possible combinations of conditions, and consistency and coverage thresholds were applied to filter out meaningful “causal recipes” for each outcome. This fsQCA analysis revealed the concurrent causality among multiple factors, offering a nuanced perspective on the complex pathways leading to chemical occupational accidents and laying a theoretical foundation for the development of targeted preventive measures.

### 2.2 Data collection

#### 2.2.1 Case selection and data source

This study focuses on chemical occupational accidents that occurred in China from January 2010 to December 2023. This time period was chosen because it is of great significance for understanding contemporary chemical safety challenges: it covers the period of rapid expansion of China’s chemical industry, along with the intensive updates of national and local safety management policies, ensuring that the sample reflects both the persistent issues and the development trends in chemical occupational safety.

To ensure data comprehensiveness and authority, case information was collected from four mutually verified sources: (1) government publications, including accident investigation reports and bulletins released via official platforms of the Ministry of Emergency Management of China (35) and provincial/municipal emergency management departments; (2) authoritative databases, including the case libraries of the Chemical Safety Education Public Service Platform (36) and the China Chemical Safety Association (37), which have compiled strictly verified accident records; (3) academic literature, extracting cases from peer-reviewed studies on chemical safety retrieved from Chinese academic databases such as CNKI (38) and Wanfang Data (39), with search keywords including “chemical” and “accidents”; (4) official media, including in-depth investigation reports from state-owned media such as Xinhua News Agency (40) and People’s Daily (41) that provide detailed accounts of accident processes and post-incident reviews.

#### 2.2.2 Data screening and validation

Rigorous screening and validation criteria were applied to ensure data quality and sample representativeness. First, only cases that could be clearly classified as general, major, or extremely major accidents are selected, with the judgment criteria being consistent with China’s “Regulations on the Reporting and Investigation of Production Safety Accidents.” Second, cases with ambiguous accident process descriptions, incomplete cause analysis, or missing liability determination were excluded to ensure information completeness. Third, cross-validation was conducted across multiple sources, and duplicate cases or those with conflicting information were removed to confirm data validity.

After data screening, cleaning, and organization, a final dataset of 154 chemical occupational accident case texts was formed. This dataset exhibits timeliness, comprehensiveness, and representativeness, providing a robust foundation for subsequent causal factors analysis and configuration paths research.

## 3 Data analysis

### 3.1 Data coding

Before formal coding, 90% of total 154 accident cases (139 cases) were randomly selected for the first round of the three-stage coding procedure (open, axial, selective coding). The remaining 10% (15 cases) were securely archived to form an independent validation subsample for rigorous theoretical saturation testing.

#### 3.1.1 Open coding

Open coding is a process of naming and categorizing phenomena through rigorous data examination. Following random sampling, the 139 selected chemical occupational accident cases underwent open coding to extract logical chains of accident causation from raw textual data. This analytical process strictly adhered to the principles of grounded theory methodology, which involves deconstructing raw data through conceptual labeling and subsequently reconstructing these conceptualizations into theoretically meaningful categories (42). Specifically, two researchers independently applied the constant comparative method to extract key statements reflecting accident causes from the reports, derive initial concepts from these statements, and refine these concepts into preliminary categories (43). Multiple rounds of cross-verification and discussion were conducted to resolve coding discrepancies, ensuring consistency in concept abstraction and category delineation. This iterative process ultimately yielded 55 preliminary categories, each assigned a unique code (see Table 1).

**Table 1:** Open coding examples.

**Case study**	**Excerpts from case documentation**	**Conceptualization**	**Preliminary categories**
Case 1: 1·7 Tank Farm Explosion, PetroChina Lanzhou Petrochemical	316#-R202 spherical tank pipeline elbow with defects, material properties below GB standards	Pipeline material not up to standard	a14-2 Design selection error
	No regular inspection or replacement plan execution	Inspection/replacement plan unexecuted	a6-1 Missing special operation procedures
	Equipment management lacked inspection and supervision	Equipment management deficient in supervision	a4-4 Failure to timely stop illegal acts
	No storage tank automatic interlock device	No storage tank interlock device	a15-2 Interlock and protection device failure
	Nearby incinerator open flame as ignition source	Open flame not isolated	a16-2 Accumulation of hazardous substances
Case 2: 3·21 Explosion, Tianjiayi Chemical (Xiangshui, Jiangsu)	Long-term illegal storage of nitrification waste causing self-ignition explosion	Illegal waste storage triggering explosion	a1-2 Illegal organization of production and construction
	Unauthorized wastewater process modification without EIA approval	Unapproved process modification	a1-1 Violation of safety facility construction procedures
	Illegal disposal of solid waste and wastewater	Illegal waste disposal	a4-2 Incomplete hazard investigation
	Nitrification waste incineration beyond EIA scope	Off-scope incineration	a16-2 Accumulation of hazardous substances
	Unqualified person appointed as legal representative	Unqualified personnel in charge	a9-2 Insufficient safety knowledge
	False safety assessment and illegal design/construction	False assessment and illegal operation	a4-1 Inadequate supervision performance
……			
Case 154: 6·18 Explosion, Shanghai Petrochemical Ethylene Glycol Plant	T450 tower bottom pump pipeline operated with defects, clamp stretching causing ethylene oxide leakage	Defective equipment causing leakage	a14-1 Equipment defects and operation with faults
	Operator emergency response delayed	Delayed emergency response	a7-1 Delayed emergency response
	Equipment severely aged after 32 years of operation	Severe equipment aging	a14-2 Material mismatch and aging corrosion
	No shutdown after anomaly detection, pressure leak sealing normalized	No shutdown after anomalies, normalized leak sealing	a4-2 Incomplete hazard investigation
	Old facility risk assessment inadequate, 60% safety investment cut	Inadequate assessment and reduced safety investment	a2-3 Disregard for abnormal operating conditions and safety red lines

#### 3.1.2 Axial coding

Axial coding, a critical phase in qualitative data analysis, is designed to identify inherently organic relationships among preliminary categories and synthesize fragmented data into coherent, conceptually elevated higher-order core categories (44). Specifically, adhering to the constant comparative method proposed by Strauss and Corbin (45), the coding process entailed three key steps: first, systematically exploring multifaceted logical connections (e.g., causal, contextual, conditional) embedded within the 55 preliminary categories to uncover latent relational structures; second, thematically clustering and subsuming preliminary categories with congruent attributes (e.g., “a1-1 violation of safety facility construction procedures,” “a1-2 illegal organization of production and construction,” and “a1-3 illegal trial production and equipment rental”) under provisionally defined main categories (e.g., “A1 ignorance of laws and regulations”); and third, conducting continuous iterative validation of these aggregated main categories through repeated comparative analysis against raw case data, ensuring their empirical grounding and accurate alignment with the nuances of real-world accident scenarios. This iterative integration condensed the 55 preliminary categories into 16 distinct main categories (see Table 2), each representing a theoretically saturated and cohesive dimension of accident causation.

**Table 2:** Axial coding: preliminary categories to main categories.

Main category	Preliminary categories
A1 Ignorance of laws and regulations	a1-1 Violation of safety facility construction procedures; a1-2 Illegal organization of production and construction; a1-3 Illegal trial production and equipment rental
A2 Deficient safety production philosophy	a2-1 Deficient safety values; a2-2 Inadequate identification and management of systemic risks; a2-3 Disregard for abnormal operating conditions and safety red lines
A3 Insufficient training, education, and safety communication	a3-1 Training content disconnected from reality; a3-2 Insufficient emergency training and drills; a3-3 Lack of risk notification and specialized training; a3-4 Formalized safety education
A4 Inadequate safety supervision and oversight	a4-1 Inadequate supervision performance; a4-2 Incomplete hazard investigation; a4-3 Inadequate management of outsourcing and supervision units; a4-4 Failure to timely stop illegal acts
A5 Deficient safety responsibility system	a5-1 Ineffective accountability; a5-2 Ambiguous job responsibilities; a5-3 Lack of contractor management; a5-4 Using contracts instead of management
A6 Inadequate documentation of safety rules and regulations	a6-1 Missing or outdated operating procedures; a6-2 Non-standard maintenance and change management; a6-3 Incomplete or unpracticed emergency response plans; a6-4 Poor enforcement of the system
A7 Lagged crisis response	a7-1 Delayed emergency response; a7-2 Blind rescue; a7-3 Insufficient emergency equipment; a7-4 Ineffective emergency drills
A8 Deficient safety awareness	a8-1 Insufficient hazard awareness; a8-2 Poor self-protection awareness; a8-3 Lack of awareness of abnormal condition handling
A9 Insufficient safety knowledge	a9-1 Insufficient understanding of hazardous material characteristics; a9-2 Failure to master safety operating procedures; a9-3 Lack of emergency knowledge
A10 Improper safety practices	a10-1 Habitual violations; a10-2 Lack of labor protection; a10-3 Inadequate safety confirmation before work
A11 Maladaptive safety mentality	a11-1 Prevalent sense of luck; a11-2 Adventurous work mentality; a11-3 Fatigue work and adventurous command
A12 Operational procedure violations	a12-1 Missing work permit; a12-2 Inadequate energy isolation and gas detection; a12-3 Violation of operating procedures; a12-4 Chaotic cross-operation management
A13 Improper command	a13-1 Improper command; a13-2 Forcing risky operations; a13-3 No safety briefing before construction; a13-4 Blind organization of construction/work
A14 Unsafe production facilities	a14-1 Equipment defects and operation with faults; a14-2 Material mismatch and aging corrosion; a14-3 Unauthorized modification and use of obsolete equipment
A15 Inadequate safety facilities	a15-1 Missing gas alarm devices; a15-2 Interlock and protection device failure; a15-3 Missing flame arresters, blind plates, or ventilation facilities
A16 Unsafe working environment	a16-1 Poor ventilation and limited space; a16-2 Accumulation of hazardous substances; a16-3 Site chaos and cross-operation disorder

#### 3.1.3 Selective coding

Selective coding, as the cornerstone final stage of grounded theory, entails identifying overarching core categories capable of integratively encapsulating all derived constructs, followed by delineating systematic relationships between these core categories and secondary categories to construct a cohesive theoretical framework (32). According to the constructivist approach advocated by Charmaz and Thornberg (46), this study operationalized the process through theoretical sampling and continuous analytical questioning, exemplified by inquiries such as “Which main categories exert the most influential role in triggering accidents?” and “In what ways do these categories interact?.” Centering on the core issue of “chemical occupational accident causation,” this study systematically abstracts, synthesizes, and integrates the previously identified 16 major categories, consolidating scattered analytical insights into a unified conceptual structure consistent with the research objective and ultimately yielding five overarching core categories (see Table 3).

**Table 3:** Selective coding: main categories to overarching core categories.

Overarching core categories	Main categories
Safety culture	A1 Ignorance of laws and regulations; A2 Deficient safety production philosophy; A3 Insufficient training, education, and safety communication
Safety management system	A4 Inadequate safety supervision and oversight; A5 Deficient safety responsibility system; A6 Inadequate documentation of safety rules and regulations; A7 Lagged crisis response
Safety capability	A8 Deficient safety awareness; A9 Insufficient safety knowledge; A10 Improper safety practices; A11 Maladaptive safety mentality
Safety behavior	A12 Operational procedure violations; A13 Improper command
Worksite condition	A14 Unsafe production facilities; A15 Inadequate safety facilities; A16 Unsafe working environment

The five overarching core categories, each representing a fundamental dimension of accident causation and encompassing logically related main categories: (1) safety culture, which captures the systemic values and norms shaping safety behavior and includes “A1 ignorance of laws and regulations,” “A2 deficient safety production philosophy,” and “A3 insufficient training, education, and safety communication”; (2) safety management system, which refers to the organizational structures and processes governing safety and covers “A4 inadequate safety supervision and oversight,” “A5 deficient safety responsibility system,” “A6 inadequate documentation of safety rules and regulations,” and “A7 lagged crisis response”; (3) safety capability, which reflects individual workers’ capacity to act safely and encompasses “A8 deficient safety awareness,” “A9 insufficient safety knowledge,” “A10 improper safety practices,” and “A11 maladaptive safety mentality”; (4) safety behavior, which denotes on-site actions that directly trigger accidents and includes “A12 operational procedure violations” and “A13 improper command”; and (5) worksite condition, which represents physical and environmental hazards and covers “A14 unsafe production facilities,” “A15 inadequate safety facilities,” and “A16 unsafe working environment.”

### 3.2 Reliability and validity assessment

Theoretical saturation testing is a critical step in grounded theory to validate the completeness of coding results and theoretical constructs; it involves halting data collection and additional coding when no new concepts or categories emerge from analysis of reserved data (47). This test serves two key purposes: (1) to enhance the accuracy and credibility of coding by minimizing subjective human bias, and (2) to ensure the resulting theoretical model exhibits high validity, with sufficient explanatory power for the phenomenon under study.

In this study, 10% of the total 154 accident cases (*n* = 15) were reserved as an independent saturation test sample, isolated from the initial coding process. After applying the same three-stage coding procedures (open, axial, selective) to these 15 cases, no new conceptual categories or inter-category relationships were identified. Furthermore, the causal factor framework derived from the main sample (*n* = 139) demonstrated strong explanatory power for the test cases, fully capturing the complex, multi-dimensional nature of chemical occupational accident causation (48). This outcome confirms the reliability of the coding process and the validity of the extracted overarching core categories.

### 3.3 Latent-active accident causation model (LA-ACM)

**Figure 2:**
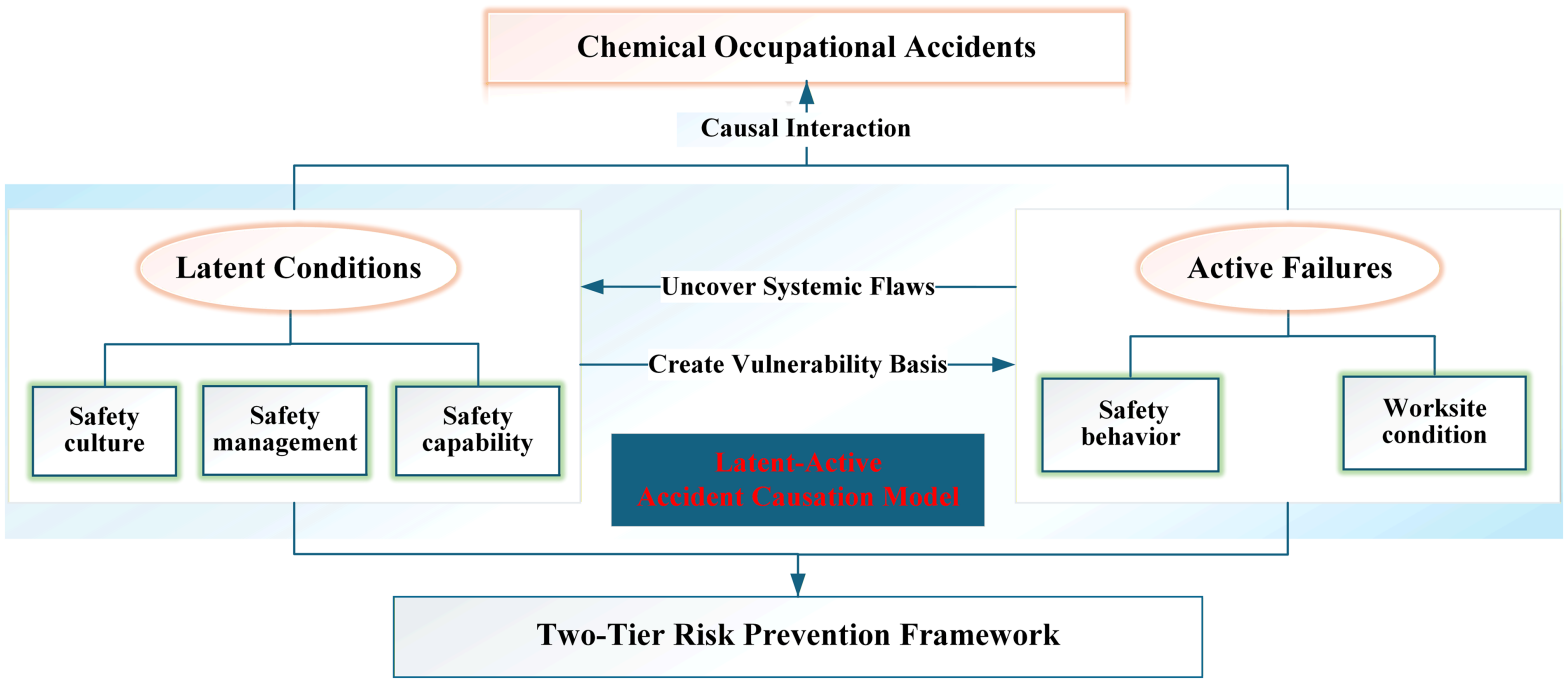
Latent-active accident causation model (LA-ACM).

Ulrich Beck’s Risk Society Theory posits that modern risks are defined by the coupling of long-term systemic risk accumulation and sudden triggering (49) —an insight that underpins the theoretical foundation of this study. Integrating findings from grounded theory’s three-stage coding, the inherent laws of chemical risk evolution, and practical risk prevention needs, this research develops the LA-ACM (see Figure 2). This model distills the multifaceted causes of chemical occupational accidents into two interrelated, hierarchical dimensions—latent conditions and active failures—thereby providing a holistic analytical framework for explaining the formation of safety system vulnerabilities and the mechanisms that trigger accidents.

Latent conditions represent the deep-seated, root causes of accidents, reflecting the long-term accumulation and diffusion of human-induced uncertainties within organizations. Encompassing three overarching core categories—safety culture, safety management system, and safety capability—this dimension exhibits distinct time-lag and cumulative characteristics. Specifically, a deficit in safety culture fosters widespread complacency toward risk among organizational members; loopholes in the safety management system (e.g., inadequate safety supervision and oversight; deficient safety responsibility system) undermine the efficacy of risk control measures; and insufficient safety capability directly limits workers’ capacity to identify hazards and respond to emergencies. These factors permeate all aspects of organizational operations, gradually eroding enterprise safety resilience over months or even years and ultimately establishing a systemically fragile foundation for accidents.

In contrast, active failures serve as immediate triggers of accidents and encompass two types of explicit, context-dependent factors: safety behavior (e.g., operational procedure violations, improper command) and worksite condition (e.g., unsafe production facilities, inadequate safety facilities, unsafe working environment). Unlike the long-term and potentially serious characteristics of latent conditions, active failures typically emerge in specific operational scenarios or emergency contexts, exhibit strong situational dependence, and manifest immediately. Acting as “triggering events,” these factors exploit vulnerabilities preconditioned by latent conditions, directly leading to unintended energy release or other accident-causing incidents.

Latent conditions and active failures are not mutually exclusive; rather, they exhibit pronounced coupling, linkage dynamics, and hierarchical progression. Latent conditions create a vulnerability basis for active failures—for instance, a deficient safety culture may normalize improper safety behavior. While accident occurrence exposes deep-seated flaws in latent conditions — for instance, an equipment-related accident may reveal long-neglected management loopholes. This forms a dynamic cycle of “accumulation of latent condition → triggering of active failure → occurrence of accidents,” underscoring that addressing only active failures cannot achieve intrinsic safety. Rather, risk governance must prioritize latent conditions to advance preventive interventions upstream.

The proposed LA-ACM transcends the reductionism of traditional accident models such as Reason’s Swiss Cheese Model (19) or the Human Factors Analysis and Classification System (HFACS) (20). These models often overemphasize singular factors, such as human error or equipment failure. In contrast, the LA-ACM adopts a systems-theoretic and configurational perspective to uncover the complex mechanisms of accident formation. The model delineates the distinct logical layers of latent conditions and active failures. It also highlights their dynamic coupling, establishing a configurational pathway framework applicable to accidents of varying severity levels. This framework provides a theoretical foundation for a two-tier prevention system that integrates latent condition rectification with active failure interception.

## 4 Configurational analysis

### 4.1 Variable measurement

#### 4.1.1 Classification of accident severity

In compliance with China’s Regulations on the Reporting and Investigation of Production Safety Accidents (State Council Decree No. 493) (50), this study categorized chemical occupational accident severity into five tiers (see Table 4) based on two core metrics: the number of fatalities (including missing persons) and direct economic losses incurred. Corresponding quantitative values were assigned to each tier to operationalize severity: extraordinarily major accidents (1.0), major accidents (0.8), relatively major accidents (0.6), ordinary accidents (0.4), and minor accidents (0.2). This classification adheres to national regulatory standards, effectively captures the gradient variation in accident consequences, and establishes an objective, policy-aligned basis for subsequent analysis of configuration paths.

**Table 4:** The operational definition and assignment of variables.

Variable category	Variable name	Classification types	Classification criteria	Assignment criteria
Accident severity	Accident grade	Minor accident	No casualties; direct economic loss < 1 million yuan	0.2
		Ordinary accident	Fatalities (including missing) < 3; injuries < 10; direct economic loss < 10 million yuan	0.4
		Relatively major accident	3 ≤ Fatalities (including missing) < 10; 10 ≤ injuries < 50; 10 million ≤ direct economic loss < 50 million yuan	0.6
		Major accident	10 ≤ Fatalities (including missing) < 30; 50 ≤ injuries < 100; 50 million ≤ direct economic loss < 100 million yuan	0.8
		Extraordinarily major accident	Fatalities (including missing) ≥ 30; injuries ≥ 100; direct economic loss ≥ 100 million yuan	1.0
Latent conditions	Safety culture	Ignorance of laws and regulations	Violations of safety facility construction procedures; illegal organization of production/construction; illegal trial production/equipment rental	Assign 1 if all three conditions are met; Assign 0.67 if two of the conditions are met; Assign 0.33 if one of the conditions is met; Assign 0 if none of the conditions are met
		Deficient safety production philosophy	Lack of safety values; inadequate systemic risk identification/management; disregard for abnormal operating conditions/safety red lines	
		Insufficient training, education, and safety communication	Training content disconnected from reality; insufficient emergency training/drills; lack of risk notification/specialized training; formalized safety education	
	Safety management system	Inadequate safety supervision and oversight	Inadequate supervision performance; incomplete hazard investigation; poor outsourcing/supervision unit management; failure to timely stop illegal acts	Assign 1 if all four conditions are met; Assign 0.75 if three of the conditions are met; Assign 0.5 if two of the conditions are met; Assign 0.25 if one of the conditions is met; Assign 0 if none of the conditions are met
		Deficient safety responsibility system	Ineffective accountability; ambiguous job responsibilities; lack of contractor management; using contracts instead of management	
		Inadequate documentation of safety rules and regulations	Missing/outdated operating procedures; non-standard maintenance/change management; incomplete/unpracticed emergency plans; poor system enforcement	
		Lagged crisis response	Delayed emergency response; blind rescue; insufficient emergency equipment; ineffective emergency drills	
	Safety capability	Deficient safety awareness	Insufficient hazard awareness; poor self-protection awareness; lack of abnormal condition handling awareness	Assign 1 if all four conditions are met; Assign 0.75 if three of the conditions are met; Assign 0.5 if two of the conditions are met; Assign 0.25 if one of the conditions is met; Assign 0 if none of the conditions are met
		Insufficient safety knowledge	Insufficient understanding of hazardous material characteristics; failure to master safety operating procedures; lack of emergency knowledge	
		Improper safety practices	Habitual violations; lack of labor protection; inadequate pre-work safety confirmation	
		Maladaptive safety mentality	Prevalent sense of luck; adventurous work mentality; fatigue work/adventurous command	
Active failures	Safety behavior	Operational procedure violations	Missing work permits; inadequate energy isolation/gas detection; violation of operating procedures; chaotic cross-operation management	Assign 1 if both conditions are met; Assign a value of 0.5 if one of the conditions is met; Assign 0 if none of the conditions are met
		Improper command	Improper command; forcing risky operations; no pre-construction safety briefings; blind organization of construction/work	
	Worksite condition	Unsafe production facilities	Equipment defects/operation with faults; material mismatch/aging corrosion; unauthorized modification/use of obsolete equipment	Assign 1 if all three conditions are met; Assign 0.67 if two of the conditions are met; Assign 0.33 if one of the conditions is met; Assign 0 if none of the conditions are met
		Inadequate safety facilities	Missing gas alarm devices; interlock/protection device failure; missing flame arresters/blind plates/ventilation facilities	
		Unsafe working environment	Poor ventilation/limited space; accumulation of hazardous substances; site chaos/cross-operation disorder	

#### 4.1.2 Measurement of conditional factors

Drawing on the grounded theory coding results of case texts, this study established an analytical framework for accident conditional factors encompassing five core dimensions: safety culture, safety management system, safety capability, safety behavior, and worksite condition. The framework decomposes the constituent sub-factors of each conditional variable in detail, clarifies their conceptual connotations, and specifies classification bases and assignment criteria (see Table 4).

Following methodological recommendations for multi-dimensional concept measurement, quantitative assignment was conducted using four-point and five-point scales under a continuous variable approach (51, 52). For example, regarding worksite condition—comprising three sub-categories—a value of 1 was assigned if all three sub-categories were present; 0.67 if two were present; 0.33 if only one was present; and 0 if none were present. Similarly, for the safety management system (comprising four sub-categories), values were assigned as follows: 1 if all four sub-categories were present; 0.75 if three were present; 0.5 if two were present; 0.25 if only one was present; and 0 if none were present. This approach ensures classification accuracy and consistency while effectively capturing the differences in the gradients of the impact of various factors on accidents.

#### 4.1.3 Assignment of variables

To ensure the objectivity and scientific rigor of quantitative scoring, this study implemented a rigorous scoring procedure aligned with the tabular classification and quantification criteria. Two researchers each assigned 20 identical case texts and conducted pre-scoring after fully grasping the scoring criteria. Upon completion of pre-scoring, the two researchers jointly reviewed and discussed the results until consensus was reached, further supplementing and refining the quantification criteria for case texts (see Table 4).

Subsequently, the researchers re-scored another 10 identical case texts and engaged in iterative discussions until full consensus on the quantification criteria was achieved. Finally, formal scoring was conducted, with any emerging questions addressed through immediate discussion to ensure the scientificity, objectivity, and consistency of the results (53).

### 4.2 Necessary condition analysis

To investigate the causal factors and their interactions underlying chemical occupational accidents of varying severity, this study grouped all cases into two categories (high-severity and low-severity) in line with the severity classification framework established in Table 4. To ensure comparability, accident cases were stratified by severity into two groups: high-severity cases (*n* = 73), comprising extraordinarily major, major, and relatively major accidents, and low-severity cases (*n* = 81), consisting of ordinary and minor accidents.

Prior to conducting fsQCA, a necessary condition analysis was performed for each conditional variable to determine whether any single variable could independently form a “necessary relationship” with the outcome (i.e., accident severity). In set-theoretic methods, a condition is typically deemed necessary if its consistency score exceeds 0.9—indicating that the outcome rarely occurs in the absence of the condition (52). Coverage is another critical metric used to assess the practical significance of a condition: low coverage implies that, while a condition may be necessary, it applies only to a limited subset of cases, meaning it is not universally prevalent across scenarios leading to the outcome (33).

Based on the coded textual data and quantified variable sets, fsQCA 3.0 software was employed to analyze the necessary conditions for chemical occupational accident severity, with findings presented in Table 5. Results showed that the consistency scores of all conditional variables fell below 0.9, indicating that no single factor alone constitutes a necessary condition for triggering chemical occupational accidents of either high or low severity. This finding underscores the complex, comprehensive, and multi-factorial nature of chemical occupational accidents, confirming that such accidents result from the synergistic interaction of multiple causal factors rather than isolated causes. Consequently, further analysis is required to unpack the interactions among factors and their combined effects—particularly to clarify how these factors exert coupled influences on the occurrence and severity of chemical occupational accidents.

**Table 5:** Necessary condition analysis results.

Condition variables	High-severity accidents		Low-severity accidents	
	Consistency	Coverage	Consistency	Coverage
Safety culture	0.8456	0.8022	0.8849	0.5754
Non-safety culture	0.4447	0.9082	0.6793	0.7060
Safety management system	0.7488	0.8184	0.7543	0.5928
Non-safety management system	0.5357	0.852	0.7451	0.6069
Safety capability	0.477	0.9303	0.7253	0.6632
Non-safety capability	0.8491	0.8235	0.8322	0.5918
Safety behavior	0.7959	0.8398	0.8724	0.529
Non-safety behavior	0.5099	0.8554	0.6378	0.7495
Worksite condition	0.6604	0.8517	0.773	0.6592
Non-worksite condition	0.6693	0.8696	0.824	0.6208

The recognition criterion for necessary conditions is typically set at a necessary consistency score of 0.9 or higher.

### 4.3 Conditional configuration analysis

Before conducting a conditional configuration analysis, it is crucial to determine the appropriate frequency and consistency thresholds to ensure the reliability of the research findings. This study uses a sample of 154 cases, which falls within the large-sample range. To ensure that each causal configuration is supported by sufficient empirical instances, the frequency threshold is set to 2 and the consistency threshold to 0.8. Configurations with consistency below 0.8 are excluded from the solution analysis.

A simplified truth table was generated using fsQCA 3.0 software, with values assigned to each configuration row based on the following criteria: configurations with consistency ≥ 0.8 and case frequency ≥ 2 were assigned a value of 1 (indicating the presence of the outcome variable), and 0 otherwise. Since no necessary conditions were identified in the preceding analysis, all conditional variables were selected as default options during the standardized analysis.

In conditional configuration analysis, three types of solutions are typically generated: simple, intermediate, and complex solutions. Simple solutions have overly simplistic structures and compromised rationality, as they incorporate all logical remainders. In contrast, complex solutions exclude all logical remainders, which may result in findings overly dependent on specific cases. Intermediate solutions, however, balance the two by incorporating only the plausible components of logical remainders, thereby ensuring the representativeness and universality of results (54).

Accordingly, this study adopts the intermediate solution as the framework for interpreting configuration results, with the simple solution used as a supplement to distinguish core conditions from marginal conditions. The configuration analysis results for the severity of chemical occupational accidents are presented in Table 6. For all configurations triggering chemical occupational accidents, the consistency of both individual solutions and the overall solution exceeded the minimum acceptable threshold of 0.75 (33). Specifically, the overall solution consistency for high-severity accidents was 0.915 with a coverage of 0.766, while that for low-severity accidents was 0.771 with a coverage of 0.838. These results indicate that the configurations identified exhibit high representativeness and universality across accidents of varying severity.

**Table 6:** Configuration of chemical occupational accident severity.

Conditional variable	High-severity configuration				Low-severity configuration		
	*H_1_*	*H_2_*	*H_3_*	*H_4_*	*L_1_*	*L_2_*	*L_3_*
Safety culture	●	●	●	●	⊗	●	⊗
Safety management system	⊗	●	●	●	⊗	●	⊗
Safety capability	⊗	—	●	⊗	—	—	⊗
Safety behavior	—	●	●	⊗	●	●	●
Worksite condition	—	●	—	⊗	⊗	●	●
Native coverage	0.426	0.438	0.345	0.306	0.412	0.525	0.405
Unique coverage	0.168	0.087	0.034	0.050	0.161	0.201	0.054
Consistency	0.925	0.951	0.957	0.990	0.937	0.829	0.866
Overall consistency	0.915				0.771		
Overall coverage	0.766				0.838		

● indicates the presence of a core condition; ⊗ indicates the absence of a core condition; ● indicates the presence of a marginal condition; ⊗ indicates the absence of a marginal condition; “—” indicates an irrelevant condition (may be present or absent).

#### 4.3.1 Configuration paths analysis of high-severity accidents

The sufficiency analysis of high-severity accidents identified four typical configurational causal paths (see Table 6). Combined with the core-marginal conditions determined via the fsQCA method and actual chemical occupational accident cases, this section further elaborates on the mechanisms of coupling and correlation among influencing factors.

1 Safety culture-driven type (Configuration H1)

Configuration H1 identifies a deficient safety culture as the core condition leading to high-severity accidents. This safety culture-driven type empirically reveals that even chemical enterprises with robust safety management system and sufficient safety capability may still experience high-severity accidents due to deficient safety culture, such as ignorance of laws and regulations, deficient safety production philosophy, and insufficient training, education, and safety communication. This configurational path underscores the critical role of safety culture in chemical enterprises, as demonstrated by the Texas explosion in the United States (55), where failures in safety culture can compromise all technical and managerial defenses (56). This configuration accounts for 42.6% of chemical production accident cases, with approximately 16.8% of cases exclusively explained by this pathway.

2 Multi-triggered type (Configuration H2)

In Configuration H2, improper safety behavior and hazardous worksite condition serve as core causal factors, supplemented by defects in the safety management system and safety culture. Its distinguishing feature lies in the synergistic amplification effect of latent conditions and active failures: When improper safety behavior—such as operators failing to wear PPE or arbitrarily removing it during work with little awareness of potential consequences (7)—coexist with hazardous worksite conditions, such as unregulated storage of hazardous substances, the marginal defense capability of the safety management system is breached (57). This combination of conditions explains approximately 54.3% of chemical production accidents, with about 6.2% of cases attributed exclusively to this path.

3 Safety capability-deficient type (Configuration H3)

In Configuration H3, insufficient safety capability is the core causal factor, combined with secondary factors such as improper safety behavior and inadequate safety management system. This configurational path reveals that despite the presence of auxiliary factors (e.g., improper safety behaviors, inadequate safety management systems, or deficient safety culture), safety capability exerts a decisive impact on chemical enterprise safety. In other words, the cumulative effect of insufficient safety capability may offset the effectiveness of other protective measures, while improper safety behavior and inadequate safety management system will further increase the probability of high-severity accidents (58). This configuration explains roughly 34.5% of chemical occupational accident cases, with about 3.4% of cases explained exclusively by this path.

4 Safety management-dominated type (Configuration H4)

Configuration H4 treats defects in the safety management system as its core condition and deficient safety culture as an auxiliary condition, resulting in a 30.6% reduction in accident-prevention effectiveness. In-depth analysis shows that “system hollowing” (e.g., inadequate safety supervision and oversight, deficient safety responsibility system) directly weakens the effectiveness of safety management system, breaches the systemic defense baseline for accident prevention (59), and becomes a fundamental causal factor in chemical occupational accidents. Safety culture plays only a secondary role under this configuration, implying that insufficient safety culture increases the likelihood of high-severity accidents under inadequate safety management system. However, such effects presuppose structural deficiencies in the safety management system and cannot, in isolation, trigger systemic safety failures.

The above configurations indicate that chemical occupational accidents are not determined by a single causal factor but by the nonlinear coupling of causal conditions (60), such as inadequate safety management system, insufficient safety capability, deficient safety culture, and hazardous worksite condition. From a practical perspective, chemical enterprises should abandon the linear “gap-filling” mindset and instead focus on interactions among elements. Particular attention should be devoted to key conditions possessing core explanatory power (notably, safety culture in H1 and the safety management system in H4), with due consideration given to the superimposed effects of complex factor configurations.

#### 4.3.2 Configuration paths analysis of low-severity accidents

Low-severity accidents are underpinned by three configurational paths, with their respective mechanisms elaborated as follows:

1 Safety behavior-induced type (Configuration L1)

Configuration L1 regards unsafe behavior as its core condition, revealing that even when an enterprise is equipped with safe worksite condition, a well-established safety management system, and a positive safety culture, isolated improper safety behavior (such as operational procedure violations or improper command) can still directly trigger low-severity accidents. It is therefore critical to standardize employees’ production and operation behaviors in the chemical industry (61). This path accounts for approximately 41.2% of the low-severity chemical occupational accident cases.

2 Worksite condition-triggered type (Configurations L2 and L3)

This category of causal paths, centered on hazardous worksite condition, consists of two sub-configurations: sub-configuration L2, characterized by the multi-dimensional coupling of hazardous worksite condition with deficient safety culture, inadequate safety management system, and improper safety behavior. This sub-configuration accounts for approximately 52.5% of the low-severity chemical occupational accidents (62). While sub-configuration L3 primarily reflects the simple superposition of hazardous worksite condition and improper safety behavior, accounting for roughly 40.5% of the low-severity chemical occupational accident cases. Furthermore, a synthesis of configurations L2 and L3 reveals that low-severity accidents may be linked to the auxiliary function of latent conditions or stem solely from active failures. Therefore, it is necessary to decompose the causes to avoid a one-size-fits-all approach to risk prevention.

### 4.4 Robustness testing

Robustness testing is an essential component of fsQCA analysis, with the primary objective of verifying and enhancing the robustness and reliability of empirical findings through systematic verification (63). Multiple methodologies exist for conducting robustness testing in fsQCA, including adjusting consistency thresholds, recalibrating anchor points, modifying variable measurement approaches, altering case frequency thresholds, using diachronic data, and switching data sources (64, 65). The robustness test results derived from these methods are compared against the initial primary analysis findings, with a focus on two critical dimensions: discrepancies in fitting parameters and the status of set relationships (66). If no significant differences emerge between the two sets of results, the empirical conclusions are deemed robust; otherwise, they fail the robustness test.

In this study, two robustness tests were implemented. First, the consistency threshold is raised from 0.8 to 0.85 while holding all other conditions constant; second, the case frequency threshold is increased from 2 to 3 with no adjustments to other parameters. A comparative analysis of the results from these two tests and the primary analysis revealed that, despite minor fluctuations in the number of identified configurational paths, key metrics, including overall solution consistency, coverage, and the distribution patterns of causal paths, varied only within a narrow, stable range. This outcome empirically confirms that the core findings concerning the configuration paths of chemical occupational accidents remain robust and reliable.

## 5 Conclusion

### 5.1 Summary

Drawing on 154 cases of chemical occupational accidents in China from 2010 to 2023, this study employs a mixed-methods research design integrating grounded theory and fsQCA to systematically explore the causal factors, configuration paths, and corresponding risk prevention mechanisms of chemical occupational accidents. The key conclusions are as follows:

First, three-stage coding in grounded theory identifies that safety culture, safety management system, and safety capability constitute the latent conditions of chemical occupational accidents, while safety behavior and worksite condition constitute active failures. Based on this, the LA-ACM was developed, which demonstrates that latent conditions undermine organizational safety resilience through long-term infiltration, whereas active failures trigger accidents against the backdrop of accumulated latent conditions—with significant coupling and interaction effects between the two. This finding transcends the limitations of traditional linear attribution frameworks and fully illustrates the dynamic evolution of chemical occupational accidents from long-term risk accumulation to immediate triggering.

Second, fsQCA analysis reveals the multi-dimensional configurational characteristics of chemical occupational accidents. High-severity accidents exhibit four typical causal configurations: safety culture-driven, safety management-dominated, safety capability-deficient, and multi-triggered. The study confirms that deficient safety culture is the core causal factor in high-severity accidents. Even with relatively robust safety management system and sufficient safety capability, a severe deficit in safety culture can still induce high-severity accidents, validating the “human-induced uncertainty diffusion” mechanism proposed in Risk Society Theory. In contrast, low-severity chemical occupational accidents primarily manifest through two pathways: safety behavior-induced type and worksite condition-triggered type. These findings not only verify the causal mechanism of multi-factor nonlinear coupling but also provide a configurational foundation for the “shift risk control upstream” strategy in the chemical industry.

Third, this study proposes a two-tier risk prevention framework, which integrates the “latent conditions rectification tier” and the “active failures interception tier” to facilitate coordinated risk governance across organizational management and frontline operations. The “latent conditions rectification tier” addresses long-term risk mitigation, encompassing the cultivation of safety culture, optimization of safety management system, and enhancement of safety capability; the “active failures interception tier” focuses on real-time intervention, including the standardization of safety behavior and the safeguarding of worksite condition. This framework provides a structured approach to risk source control and layered prevention, offering both theoretical and practical implications for safety governance in the chemical industry.

### 5.2 Policy implications

Based on the LA-ACM model and configurational path analysis results, this study proposes a two-tier risk prevention framework for the chemical industry, which offers risk management strategies adaptable to diverse national and operational contexts.

First, a “trinity” governance system for latent condition rectification should be established through three interconnected strategies to enhance organizational safety resilience: (1) Regarding safety culture cultivation, enterprises should embed safety concepts into corporate strategy through compliance training, case-based warning education, and leadership safety demonstration (67), while fostering a “safety-first” organizational atmosphere by integrating safety performance into both employee and managerial appraisal systems. For instance, enterprises can learn from the German chemical company Badische Anilin- und Soda-Fabrik (BASF) by deeply integrating safety leadership into its global operations (68). (2) For safety management system optimization, chemical enterprises need to implement precision supervision, establish a full-life-cycle safety risk control mechanism covering design, operation, and decommissioning stages, and strengthen targeted audits and hierarchical control measures for high-risk chemical enterprises (69). (3) For safety capability enhancement, establish corresponding job competency standards, and utilize VR/AR-based training to enhance employees’ risk identification and emergency response capabilities (70), as demonstrated by Brazil’s National Industrial Training Service (SENAI) partnering with DNV to co-develop a VR emergency drill platform (71).

Second, an intelligent prevention system for active failure interception could be constructed, aiming to achieve accident interception by means of real-time monitoring and rapid intervention: (1) Industrial Internet and AI technologies are leveraged to create a dynamic monitoring and early-warning system for production facilities, enabling real-time risk surveillance of equipment and working environments (72). (2) Employ real-time monitoring and automated compliance checks for confined space operations through intelligent video surveillance systems and tiered risk protocols (73), such as learning from Singapore’s Jurong Island Chemical Park model. (3) A four-level hazard inspection mechanism (“daily check – mutual check – weekly check – monthly check”) is implemented to ensure comprehensive identification and timely rectification of worksite condition defects (74).

Third, to enhance the effectiveness of the two-tier risk prevention framework, a collaborative risk governance ecosystem with multiple stakeholders needs to be established to break down governance barriers: (1) A cooperative regulatory mechanism between government and enterprises should be established to enable cross-organizational data connectivity and precise risk profiling, leveraging proven models such as the American Chemistry Council’s “Responsible Care” initiative (75), which in turn can support the development of a global joint prevention and control mechanism that transcends administrative and geographical boundaries. (2) Establish a cross-departmental safety cooperation community in chemical parks (76), integrating resources from emergency management, industry, and information technology, as well as ecological environment departments, to implement unified risk information sharing and precise law enforcement. (3) Cultivate a market-oriented safety service system by introducing third-party professional assessment agencies for independent risk evaluations, creating a governance framework involving government, enterprises, industry associations, and the public.

This study, while contextualized in the chemical industry, is underpinned by a logical framework with generalizable relevance to safety regulation in high-risk sectors such as mining and construction. Empirical evidence indicates that accidents across these domains share a common etiology—the complex interaction between latent conditions and active failures. For example, mining incidents often arise from the confluence of equipment malfunctions (e.g., ventilation failure) and systemic gaps in emergency preparedness (77), while construction hazards frequently stem from interactions between procedural violations (e.g., unauthorized aerial work) and deficiencies in subcontractor oversight (78). In light of this common mechanistic basis, the “trinity” governance system proposed in this study targets root causes such as weak safety culture and managerial loopholes; the intelligent prevention system enables real-time risk monitoring and interception; and the multi-stakeholder framework enhances regulatory precision and effectiveness by breaking down institutional silos. Together, these components form an integrated, systematic strategy that addresses both deep-seated and immediate risk factors, offering a transferable model for safety governance across high-risk industries.

### 5.3 Limitations

This study has several limitations that warrant further investigation. First, although the research sample predominantly consists of chemical occupational accident cases in China from 2010 to 2023, it does not differentiate among specific subsectors. Sectors such as petrochemicals, coal chemicals, and hazardous chemical storage and transportation are not separately analyzed. This lack of subsector-specific analysis may constrain the generalizability of the findings across distinct industrial segments. Second, external contextual factors, including temporal dynamics and regional disparities, were not incorporated into the analysis. The dynamic mechanisms of accident causation also remain underexplored.

Future research may advance along two dimensions. On one hand, expanding the sample coverage to study process types and industrial chain segments in depth would help construct a more comprehensive map of chemical occupational safety risks. On the other hand, cross-country comparative studies are needed, especially those involving representative chemical industry leaders such as the United States and Germany. These studies can help explore variations in accident configurational paths across diverse institutional and industrial contexts, thereby providing theoretical support for global chemical occupational safety governance (79).

## Data Availability

The raw data supporting the conclusions of this article will be made available by the authors, without undue reservation.
